# Barriers to Screening for Gestational Diabetes Mellitus in New Zealand Following the Introduction of Universal Screening Recommendations

**DOI:** 10.1089/whr.2021.0149

**Published:** 2022-05-02

**Authors:** Lynne Chepulis, Valentina Papa, Brittany Morison, Shemana Cassim, Ruth Martis

**Affiliations:** ^1^ Medical Research Centre, Te Huataki Waiora School of Health, University of Waikato, Hamilton, New Zealand.; ^2^ The Liggins Institute, The University of Auckland, Auckland, New Zealand.

**Keywords:** gestational diabetes mellitus, clinical guidelines, diabetes screening, pregnant women, thematic analysis, barriers to screening

## Abstract

**Background:**

In 2014 the New Zealand Ministry of Health implemented a universal program of screening for gestational diabetes mellitus (GDM) in pregnancy; however, data suggest that only half of all women are being screening according to the guidelines. This study aimed to explore women's views and experiences of GDM screening and to determine what the main screening barriers are.

**Methods:**

Eighteen women were recruited from the Waikato region of New Zealand, who were either pregnant (>28 weeks of gestation) or had given birth in the last 6 months. These women participated in a semi-structured interview about their experience of GDM screening and the transcripts were thematically analyzed. Of these women, 14 had been screened for gestational diabetes (three were screened late) and four had not been screened at all.

**Results:**

Multiple barriers to screening for GDM were identified, with two overarching themes of “confusion, concerns, and access to information for screening,” and “challenges to accessing and completing the screening test.” Specific barriers included the preference of risk-based assessments for GDM by their leading health professional (usually a registered midwife); negative perceptions of “sugar drink test”; needing time off work and childcare; travel costs for rural women; previous negative screening experiences; and reduced health literacy.

**Conclusion:**

There appear to be both woman-, midwife-, and system-level barriers to screening for GDM. While screening is ultimately a woman's choice, there does appear to be capacity to increase screening rates by improving awareness of the updated guidelines, and making the test environment more accessible and comfortable.

## Introduction

Gestational diabetes mellitus (GDM) is a state of glucose intolerance that is first detected during pregnancy but usually resolves during the postpartum period.^1^ Women diagnosed with GDM may develop serious health complications resulting in short and long-term health risks for the pregnant woman and her baby.^2,3^ Further, women with a previous diagnosis of GDM have an adjusted risk of developing type 2 diabetes (T2D) that is 17.9 higher than that of women without a history of GDM during the 3–6 years postbirth.^4^

In New Zealand, the risk of developing GDM is increasing, with ∼8% of pregnant women being diagnosed annually with GDM.^5^ Incidence rates are higher in those of Māori (the indigenous people of New Zealand) and Pacific descent,^6^ and earlier New Zealand data show that rates of pregnancy complications for women with GDM have increased from 1.3% in 2001 to 4.9% in 2012.^7^

As such, in 2014, New Zealand developed diabetes in pregnancy (DiP) screening guidelines that recommends universal screening of GDM of all pregnant women, irrespective of risk factors.^5^ The guidelines recommend a two-step screening process, including a glycated hemoglobin (HbA1c) test at before 20 weeks gestation to exclude undiagnosed pre-existing diabetes.

This should then be followed by a 1-hour glucose challenge test (Polycose or GCT) at 24–28 weeks gestation for pregnant women who are assessed to be at low risk for GDM and/or a 2-hour oral glucose tolerance test (OGTT) for pregnant women who are assessed to be at high risk of developing GDM or who have a positive Polycose result.^5^ These tests are routinely performed at pathology laboratories throughout the country and women may be referred for testing by their midwife, General practitioner, obstetrician, or other clinician. Most midwifery care in New Zealand (including DiP screening) is undertaken by midwives.^8^

In a recent review of these DiP screening in nearly 6000 women who birthed in the Waikato region during 2017 and 2018, screening for GDM was shown to be suboptimal,^9,10^ despite the current guidelines. For example, while more than 90% of women completed an HbA1c test, only three quarters (77.1%) of women went on to be screened for GDM.

GDM screening was even lower for indigenous Māori women in New Zealand (64.1%), and across all ethnic groups 20%–30% of those screened did so late after 28 weeks gestation.^10^

It is currently unknown whether this may be due to midwives preferring to use risk-based screening criteria rather than universal screening,^11^ issues with the OGTT test (*e.g.,* the propensity for nausea^12,13^) or because the two-step screening process may be obstructive by placing additional demands on both midwife and the pregnant woman.^14^ Given the importance of recognizing GDM early in pregnancy, and New Zealand's recommendation for universal screening it is important to better understand why screening is not occurring and/or why it is occurring very late in pregnancy. Thus, the aim of this study was to explore the women-centric views and experience of GDM screening in New Zealand so as to better understand the barriers to screening.

## Methods

### Design, ethics, and procedure

Semi-structured interviews were carried out by V.P. between December 2019 and January 2020 with women who were and were not screened for GDM. An interview guide created by the research team and based on broad themes related to screening choices (Supplementary File S1) was used to guide/prompt the facilitation of the interview and included exploring women's views and experiences of GDM screening.

Participants were recruited by posting on two social media groups—one for women with GDM in New Zealand and one for pregnant and postnatal women who used a large regional free-standing birthing center. In addition, women were recruited directly from the DiP clinic at the Waikato District Health Board (WDHB). In all instances, study information was posted/provided to women in clinic, along with contact information for L.C. and V.P. for any women who were interested to be involved. After initial email enquiries about the study, those who opted to participate arranged an interview with V.P.; either in person at their home or over the phone, with interviews lasting 30–45 minutes.

We accepted all women who wanted to participate, which included a range of age, ethnicity, and screening choices. As we had good representation of women who were and were not screened, no additional targeted screening was required.

The study was approved by the Waikato Human Research Ethics Committee HREC(Health) 2019#42.

### Participants

Women were invited to participate if they were either pregnant (>28 weeks of gestation) or had given birth in the last 6 months, were living in the Waikato region and able to communicate in English. The 28-week gestation point was chosen as screening for GDM should occur before 28 weeks according to the guidelines.^5^

Eighteen women (including 5 Māori women, 10 New Zealand European [NZE] women, and 3 women of other ethnicity) with an age range of 25–37 years (mean 31.4; standard deviation 4.3) met the inclusion criteria (Table 1). All participants had been screened for pre-gestational diabetes early in their pregnancy *via* HbA1c. Of the 18 women, 11 (61%) who had been screened for GDM within the recommended screening gestation, 3 (17%) women were screened late (after 28 weeks gestation) and 4 (22%) women had not been screened at all for GDM. Six women (33%) were primigravida or in post-partum period with their first child (Table 1). Seventeen women in this study had a registered self-employed midwife for their pregnancy care and one woman had engaged a private obstetrician.

**Table 1.** Demographic Characteristics of Participating Women (*n* = 18)

Maternal characteristics, *n* (%)	
Age^a^ (range, years)	25–37
Age^a^ (mean, years) (SD)	31.4 (4.3)
Primigravida	6 (33)
Multigravida	12 (67)
Previous GDM	3 (17)
Screened between 24 and 28/40	11 (61)
Screened late (after 28/40)	3 (17)
Never screened	4 (22)
Ethnicity, *n* (%)	
NZ born European	10 (56)
Māori	5 (28)
Other	3 (17)

^a^ There was no age available for two women *n* = 16.

GDM, gestational diabetes mellitus; SD, standard deviation.

### Data Analysis

The interviews were all recorded *via* a digital audio recorder with additional field notes made during the interview. Audio recordings were transcribed and anonymized by V.P. The transcripts and field notes were thematically analyzed^15^ and the reporting of this study is based on the Consolidated Criteria for Reporting Qualitative Research studies guidelines.^16^

Thematic analysis^15^ was initially carried out by the two researchers (V.P. and B.M.) independently, and then together. The broad themes and findings were then shared, discussed, and debated with the broader team as a way of ensuring a rigorous and reflexive analysis process. This process involved a thematic analysis, comparison, and robust discussions and reanalyzing occurred until saturation was reached. The team members who carried out the analysis of data comprised a combination of early career and mid to late career researchers, who all collectively contributed to the analysis process.

## Results

While the initial aim of the study was to explore the experiences of women in relation to GDM screening, the interview data revealed multiple barriers to screening for GDM experienced by the women in this study. These were grouped into two overarching, yet interrelated themes based on barriers relating to (1) confusion, concerns and access to information for GDM screening, and (2) challenges to accessing and completing the OGTT screening test.

### Confusion, concerns, and access to information for GDM screening

#### Decision based on choice

Many participants expressed confusion and concerns about whether screening for GDM was actually required. Some were advised that all women should be screened for GDM, while others were given the opportunity to make the choice themselves. Those in the latter group suggested that being able to make this decision themselves allowed them to feel “in control” of their pregnancy.

“I felt very empowered that I could make those decisions I wanted to make, and I did not feel at any point like I was forced to do something… I feel that for a lot of people they do choose to do that diabetes test purely because everyone around them does it and don't know that actually you can choose not to” (Participant 8).

However, many stated that they would have preferred stronger input from their midwife to guide this decision making:

“To me it was optional, but I think maybe if it was said that this test is something that ‘you get done’ rather than ‘you don't have to get it done if you don't want to’… like obviously it's optional but to me it was more like ‘you don't have to get it done’ so I was like umm okaaaay? So, should I get it done then?” (Participant 5).

#### Decision based on risk factors and experience

The presence of possible GDM risk factors, and/or experiences with GDM during previous pregnancies were also shown to influence whether women were screened for GDM in their most recent pregnancy. Women identified, for example, that their midwife was more likely to recommend screening if they were aged over 35 years, overweight/obese, had a family history of diabetes, and/or had GDM in previous pregnancies. Similarly, other participants stated that if their midwife did not believe them to be at risk of developing GDM, then screening was deemed unnecessary and it was not strongly recommended. Therein, these women were not provided an opportunity to get more information relating to GDM screening.

However, several of these women made the personal decision to screen anyway, and they were then diagnosed with GDM. This may also serve as a system barrier that prevented the midwives from offering these women screening, and thus preventing these women from getting screened.

“She wasn't worried about me because I was quite low risk. So she was pretty shocked when she found out that I did have it (GDM), as I still went to have the test” (Participant 13).

“I asked the midwife if I had to do the test with the drink and she said no because I was low risk, but I did it anyway… and then I developed GDM” (Participant 17).

#### A lack of information

Participants indicated that irrespective of whether they were pregnant for the first time or had prior pregnancies, they did not receive any information from their midwife or obstetrician about what GDM is, or how it could affect them or their child during pregnancy and beyond. Several suggested that it would have been helpful to have this information so as to better inform their screening decisions.

“I didn't know much about it so yeah it would have been good to have that extra information from my doctor and know why you have to get the test and why and what it actually is” (Participant 2).

“I've been trusting my midwife so all that she says I've just been going with it… She did not talk about this. I think I'm lucky that I got a good one … or is this compulsory for the midwives to tell you [about the screening]? Well, she's a good one anyways” (Participant 6).

Women with more than one prior pregnancy thought that their midwife assumed that they were familiar with the screening guidelines, and thus did not give them any information about GDM.

“It's your fifth baby, you know what you are doing, my midwife said” (Participant 14).

“I feel that I'm expected to know everything because this is not my first pregnancy. They did not explain again about diabetes in pregnancy” (Participant 7).

#### Clear communication as an enabler

In contrast, other women discussed that clear communication by their midwife about GDM was beneficial and highly valuable to them to support their decision about why the test was important to do.

“Yeah so my midwife went into detail about diabetes in general (T1D and T2D) and then she went into GDM to break it down to me. And she did go into a lot of detail about it, so yeah I understood once she explained it to me and then I had the test” (Participant 10).

### Challenges to accessing and completing the OGTT screening test

#### (Mis)information and perception of test

Some women made the decision to screen, or not, based on what they knew about the screening test. The concerns including overloading the baby with sugar and its perceived potential long-term effects for the baby, but also what this large amount of glucose (sugar) would do to their own metabolism. This was a factor that led to some women choosing to not be screened.

“I don't like sugar, we are quite a natural household. If I'm not in any of those risk factors, then why do I need to drink this sugary drink?” (Participant 18).

“For me it is definitely the right choice to not do it and if I had more children I still wouldn't do the screening … all the sugar and the chemicals that there are in that drink…I just think that putting that much sugar in such a short period of time isn't healthy and isn't a good thing… Especially, when sugar is meant to be so bad for your baby” (Participant 13).

#### A need for published, accessible information

However, most women agreed about the importance of GDM screening and that the long-term impact of GDM for the mother and baby should be better published and explained by their midwife or obstetrician. Women also suggested that TV ads and social media campaigns could be used to increase women's GDM awareness, including materials in languages other than English.

“I haven't seen it advertised anywhere; I just know that it is a thing. Umm but I think that if you could see… well you know they've got adds on TV for like babies with whooping cough and stuff like that. So, if you could actually see the effects that it could have on babies and children then it might end up being something that we know about?” (Participant 17).

“It's not really advertised is it? I haven't seen it around, not a lot… not that it has caught my eye…Another thing that was quite useful in Australia that wasn't provided here it was just a pamphlet with all the dates that of things you had to get done. So at these many weeks you have to get this. So it was just a list with key dates. So that was useful for me as a first mum” (Participant 16).

#### Cost and time constraints

Several participants also identified challenges relating to the screening test, particularly highlighting issues of accessing and ability to complete the screening test. Women living in rural areas identified that the costs of traveling to the laboratory and paying for childcare were significant barriers to accessing the screening test.

“The Laboratory is in Huntly, so 18 kms away. To get to an appointment is a bit of a pain also… and petrol costs… If it is something to do with the kids then I will go into town, but if it's something to do with me then I might have to put it off for a little bit. When it takes longer, I have trouble with childcare and no money for it anyways” (Participant 14).

Finding flexible childcare options was a further challenge, particularly if the laboratory was running late. This meant not being able to attend the clinic for the GDM screening test or deciding not to screen in a subsequent pregnancy. Similarly, the time associated with the OGTT test itself was a barrier, particularly for those who needed to take time off work to complete the test. This was made more difficult by the fact that the test needed to be booked in advance, which made it inflexible and more challenging to arrange for time off work.

“… The only issue is that it took a few hours and I had to get time off work for the booked appointment. That was the only thing that might've discouraged me to get it done. And to be honest, I don't know if I will get it done again because umm like I don't know, I will have to think about it a bit more… Because, just because it's not a very nice test to do… it takes a few hours” (Participant 5).

Other women suggested that they would be more likely to be screened if it only included the 1-hour test:

“I will definitely only do the one-hour one though, unless there was a reason to do the two-hour one… The choice of the one-hour one is much better, but if it shows that there could be a possibility (of developing GDM) then I will do the two hours one” (Participant 16).

“It was easier to do the one hour one because the two hours one didn't appeal” (Participant 9).

#### Previous negative experiences and environment

Multiple participants also discussed negative experiences around the GDM screening tests that influenced their decision to screen or not to screen for GDM in their current and/or future pregnancies. In particular, women who had either personal experience of, or had heard of others vomiting or having nausea during the OGTT.

“In my last pregnancy I had to do it [GDM screening] 4 times because I vomited each time. I was really unwell and vomited each time after having that drink.… I don't really want to put myself through that again” (Participant 17).

“Well the test itself is not the nicest thing to do so I don't know how you would improve that… The drink is disgusting like yeah it's definitely is for a reason (the test) but… you know … I don't know…” (Participant 5).

Finally, the laboratory test environment itself was also shown to be an off-putting one to many. When participants were asked what they would like to change about the GDM screening process or what would make their experience less pleasant, some suggested having more comfortable seats for pregnant women in the waiting area or childcare play areas.

“…and if I was allowed to have the drink and come home or have it in a more comfortable place. Because it's not very nice going in when you are nearly 30 weeks pregnant and sitting on an uncomfortable seat in a blood lab” (Participant 17).

“Umm maybe the waiting area, I know you can't leave but the chairs were so uncomfortable, so that made it harder for me.” (Participant 10).

“It would be handy if you could take your kids along to the test… I have trouble finding childcare, maybe the medical centres or labs could make playgrounds, it is too hard to entertain them” (Participant 2).

Overall barriers relating to accessing and completing the screening test related to the distance to the clinic, duration of the screening test, and conditions in the clinic and waiting room.

## Discussion

This study aimed to explore women's experiences of screening for GDM and to identify possible barriers. Findings indicated that the barriers identified by women in New Zealand were based around information for GDM screening and the screening test itself.

One key point of discussion that was observed was that the decision to screen was often largely dependent on the views of the midwife. This is consistent with previous research^11,17^ with New Zealand data suggesting that midwives may be following the earlier guidance of the New Zealand College of Midwives,^18,19^ the professional body that represents ∼90% of the practicing midwifery workforce. Specifically, in November 1996 and updated in 2002, New Zealand College of Midwives ratified a DiP consensus statement that indicated there were no data to support routine universal GDM screening for pregnant women and that only women who met one or more risk factors were encouraged to screen for GDM.^19^

Indeed, international data are conflicting with some arguing that there is insufficient evidence for risk-based GDM screening^20^ while other studies demonstrate that risk-based screening could lead to up to one-third of women with GDM being missed,^21^ which could have clinical consequences for both mother and baby. It has been anecdotally reported that the 2014 guidelines for universal screening were never widely disseminated to the midwifery community, and clearly this may need to be addressed, along with education for the need of universal screening for all women.

Regardless of the midwives' viewpoint, the participants in this study identified that having a meaningful relationship and effective communications with their midwife was a significant factor in making their decision to screen for GDM. This indicates the importance for any health care provider to be diligent in providing evidenced-based information and an awareness of how they can influence a women's decision-making process.^22,23^

In concordance with this, the International Confederation of Midwives (ICM) and NZCOM both agree with the publicized ICM statement (2014) that “Midwives provide women with appropriate information and advice in a way that promotes participation and enhances informed decision making.”^24^ Similar statements are also noted by the Royal Australian and New Zealand College of Obstetricians and Gynaecologists.^25^

Women in this study also identified the limited access to clear and simple information regarding GDM and screening tests as a barrier. While some midwives provided sufficient verbal information about GDM and the screening tests, women wanted additional simple information in print to not only remind themselves about the information heard but also to share the information with their partners and support network. Studies researching health literacy have shown many health professionals do often overestimate the level of health literacy of their clients,^26,27^ and we suggest that a lack of tailored communication (verbal and written) may lead to nonadherence to the recommended screening tests for GDM even if recommended by the midwife.

A few participants also suggested that a TV campaign about the seriousness of GDM and when and how screening tests are required may encourage the uptake of screening for GDM. This suggests that there are women who are likely actively seeking out information about GDM screening, but that the resources available in New Zealand may be lacking and/or are not as accessible as they could be.

Preconceived assumptions also posed a barrier to GDM screening, with several participants suggesting that their midwives assumed that they had retained prior information about GDM screening tests and offered little new information. This meant some women thought GDM screening tests were not important or necessary for the current pregnancy, particularly if they had not experienced GDM in any earlier pregnancies. Interventions to overcome similar barriers in other countries include supporting health literacy of women by identifying the pre-existing knowledge of GDM, confirming that the information was understood, reinforcing the information using multiple modalities including YouTube clips, visually inviting pamphlets, and involving support people.^28^

Furthermore, cultural appropriate resources have been identified as being an important aspect of supporting GDM screening, as the literature suggests that migrant groups can be less likely to be screened.^29^ This needs to be evaluated in the New Zealand context,^30^ though any health promotion program for GDM in New Zealand should also include materials in Māori, Pacific, and Asian languages, as these groups comprise the largest proportion of GDM cases in this country.^5^

Lastly, participants in the study shared their perception of the challenges to accessing and completing the screening test. For women who had undergone a GDM screening test in a previous pregnancy, knowledge of factors such as travel and childcare costs, length of the test, the laboratory environment, and concern about the possibility of nausea and vomiting all influenced the women's decision making. Similarly, previous research demonstrates that access to testing facilitates and women's negative views of the OGTT itself was affecting decision making and thus limiting the uptake of GDM screening.^31,32^

In particular, Reid et al. noted that there was an insufficient funding model for rural and remote rural midwifery in New Zealand,^31^ and that this affects the level of maternity care in these regions.^33^ Further, these barriers around access to the test have been reported elsewhere (particularly in low income settings),^34,35^ and as such, we suggest that there is an urgent need for further research to evaluate how additional factors such as provisions of petrol vouchers, improved testing facility environments (including childcare), and improved access to diagnostic facilities for rural women could lead to an increased uptake of screening.

This study had many strengths, including the fact that participating women were from a diverse ethnic and geographical background within the Waikato region, including rural and urban. Furthermore, participating women were of diverse parity, in terms of their experience of a GDM diagnosis, and of opting to have screening test.

Possible limitations included the fact that only women who were fluent in English were eligible for this study. It is unclear whether women who were not fluent in English would have elicited different or further barriers to GDM screening, thus this could be an avenue for future research. Moreover, given that this study focused on participants based in the Waikato, future research could also investigate barriers to GDM screening as experienced by women in other regions in New Zealand.

## Conclusions

This study identified a number of barriers for pregnant women to universal screening for GDM that could be used to provide guidance to support future screening initiatives. Dissemination and acceptance of any new guidelines is essential, and specific, ongoing dissemination of the current 2014 Ministry of Heath guidelines may still be warranted. Clear, concise communication between the midwife and woman is also essential to enable informed decision making about GDM screening, and the experiences of any previous pregnancies should not be considered in isolation. Lastly, research is required to determine whether GDM screening rates could be improved by reducing the barriers associated with transport, childcare, the testing facilities, and rural access. Laboratories may want to consider providing comfortable chairs and play equipment for preschool children. All of the above should also be evaluated in New Zealand women for who English is not their first language.

## Supplementary Material

Supplemental data

## Abbreviations Used

DiP: diabetes in pregnancy
GDM: gestational diabetes mellitus
HbA1c: glycated hemoglobin
HREC: Human Research Ethics Committee
ICM: International Confederation of Midwives
NZCOM: New Zealand College of Midwives
NZE: New Zealand European
OGTT: oral glucose tolerance test
SD: standard deviation
T2D: type 2 diabetes
WDHB: Waikato District Health Board
