# Supplementary material for: Barriers to Screening for Gestational Diabetes Mellitus in New Zealand Following the Introduction of Universal Screening Recommendations
Source: Womens Health Rep (New Rochelle). 2022 May 2;3(1):465–72. doi: 10.1089/whr.2021.0149 (PMC9148651; doi:10.1089/whr.2021.0149)
Supplement: Supplemental data [file Suppl_DataS1.docx]

**Diabetes in Pregnancy Interview Guide:**

20 - 40 women (> 28w gestation or who have given birth in the last year) in the Waikato region

**You do not have to ask all the questions. Let the participant lead the conversation. However, if the conversation stalls or goes off on a tangent, you could use these questions as prompts.*

(1st meeting) Introduction - **Whanaungatanga**

- Thank participant for their time and agreeing to participate
- Ask about karakia (cultural prayer to open)
- Introduce self/background including where you’re from
- Respond to participant’s own introduction
- Answer questions about yourself
- Ask if it’s OK to start the interview
- Explain aims of research and interview
- Verbally go through participant information sheet, and answer any questions/clarify any doubts
- Give participant information sheet and consent form (or verbal consent based on situation)
- Consent for turning Dictaphone on

**Could you please tell me about what you know about screening for diabetes in pregnancy**

- Are you aware that there is free testing for DiP available in NZ ? (< 20w and at 24-28w)
- At what point did you get a midwife / LMC during this pregnancy? Did she inform you about these tests?
- If no midwife, or late to sign up with one, did you see a GP during pregnancy? Did S/he advise you about the availability of screening for DiP?
- Do you know why the DiP screening tests are recommended during pregnancy?

**Barriers and Enablers to screening (**Assuming participant was aware of the screening)

- Are there factors / issues that made it difficult for you to get the screening done at the times you wanted to do it? (ie exploring reasons for maybe late testing, or lack of one test etc) –
- If so, what where these?

DO NOT specifically prompt, but looking for factors like needing to take time off work, the length of time needed for the GTT (60 or 90 mins), childcare issues, distance from laboratory, cost to get into town etc.

- Were you aware that there are two tests available during pregnancy? If YES, then were there reasons why the you chose to not do the second test? (the second test is the one that most women do not do, but this is the test for GDM). Exploring the possible idea that women have the first test, get a normal result and think that all is ok, without knowing that the second test is important and standalone). Did the midwife recommend the first / second test?
- What are some of the factors that made it easier for you to complete DiP screening?
- If the woman did not screen for DiP, explore (without leading) whether this choice was informed by the provision of information or resources, was a self-choice, midwife-recommended choice etc,

**Access to Resources**

- Were you provided with any information to support your decision about whether you wanted to go ahead with screening for DiP?
- Were you given the opportunity to discuss any concerns that you may have about screening with your midwife / LMC?
- Are there any aspects that made the process easier for you and your whanau?
- Perceived improvements? (What could be done that would have made it easier for them to have been screened at the appropriate time?)

**Who were some of the key people that helped you along this process?**

**What advice would you give to someone else considering screening for DiP?**

- Explore these topic(s), including the reasoning behind the thoughts/opinions

**Is there anything you would like to change about the way the screening for DiP is advertised / offered to women in NZ?**

Is there anything you would like to bring up or think should have been discussed?

- Do you have any further questions about this study?
- Do they want to close with a karakia?

***Reminder***

- Address/contact details to send through summary report of research - *if required*

_______________________________________________________________________________________________________________________________________________________________________________________________________________________________________________________________

***Thank you so much for your time***
